# Cardiovascular Disease Risk Factors and Health Outcomes Among American Indians in Oklahoma: the THRIVE Study

**DOI:** 10.1007/s40615-016-0310-4

**Published:** 2016-12-06

**Authors:** Valarie Blue Bird Jernigan, Marianna Wetherill, Jordan Hearod, Tvli Jacob, Alicia L. Salvatore, Tamela Cannady, Mandy Grammar, Joy Standridge, Jill Fox, Jennifer Spiegel, AnDina Wiley, Carolyn Noonan, Dedra Buchwald

**Affiliations:** 1 College of Public Health, University of Oklahoma Health Sciences Center, 4502 E 41st St., Tulsa, OK 74105 USA; 2 Choctaw Nation of Oklahoma Health Services Authority, One Choctaw Way, Talihina, OK 74571 USA; 3 Chickasaw Nation Nutrition Services Department, 518 E. Arlington, Ada, OK 74820 USA; 4 Initiative for Research and Education to Advance Community Health (IREACH), Washington State University, 1100 Olive Way, Suite 1200, Seattle, WA 98101 USA

**Keywords:** American Indian, Native American, Cardiovascular disease, Diabetes, Obesity, Vegetable and fruit intake, Community-based participatory research

## Abstract

**Introduction:**

Limited available data document higher prevalences of cardiovascular disease (CVD) risk factors and health outcomes among American Indians (AIs) compared to other racial/ethnic groups.

**Methods:**

As part of a randomized control trial to improve tribal food and physical activity environments, our tribal-academic partnership surveyed a cross-sectional sample of American Indian adults (*n* = 513) to assess the prevalence of type 2 diabetes, obesity, hypertension, tobacco use, physical activity, and vegetable and fruit intake. Surveys were collected from April through May 2015. We used logistic regression to examine the association between CVD-related risk factors and health outcomes.

**Results:**

The prevalence of CVD-related outcomes was high, ranging from 25% for diabetes to 75% for low vegetable intake. The prevalence of diabetes, obesity, and hypertension tended to be higher among participants with any tobacco use compared to no tobacco use, but findings were not statistically significant. The prevalence of diabetes (prevalence ratio 2.1, 95% CI 1.4–3.2) and obesity (prevalence ratio 1.5, 95% CI 1.2–1.8) was higher among participants with low physical activity levels compared to recommended physical activity levels.

**Conclusions:**

CVD risk factors and health outcomes persist among American Indians even as some risks (e.g., smoking) appear to be stabilizing or even declining in the general US population. Efforts to include American Indians in national health surveys, implement broad reaching environmental and policy interventions, and address the social determinants of health are critical to the elimination of these disparities.

## Introduction

Although data for American Indian (AI) populations are more limited than for other populations, available data indicate significant and widening disparities in cardiovascular disease (CVD) risk factors such as tobacco use, physical activity, vegetable and fruit intake, health outcomes such as type 2 diabetes, obesity, and hypertension. Our analysis of Behavioral Risk Factor Surveillance Survey (BRFSS) data, one of only a few national health surveys that collects data on AIs, found that from 1996 to 2006, the adjusted prevalence of obesity among AI adults increased by one quarter, from 25 to 31%, while diabetes increased by 27%, from 6.7 to 8.5% [1]. Hypertension increased by 5%, from 28.1 to 29.5% [1]. During the same time period, we found no meaningful improvements in smoking behaviors, physical activity, or fruit or vegetable intake [1].

In Oklahoma, currently ranked 45th in the nation with regard to its health outcomes [2], BRFSS data reveal that adult AIs are even more likely than whites to be obese (42 vs. 31%) and to have diabetes (15 vs. 10%) [3]. More than 60% of AI youth in Oklahoma are overweight [4], and 22% are obese [5], even though AI youths are at least as concerned about their weight as non-Hispanic whites [6]. Food insecurity, the limited or uncertain availability of healthy foods, a condition highly associated with obesity, diabetes, and hypertension [7], is three times more common among AIs than whites (21.3 vs. 7.3%, respectively) [8]. Finally, less than half of AI adults in Oklahoma meet current physical activity guidelines [9].

Tribal nations have implemented a number of programs to prevent and address CVD and diabetes, primarily by offering health education to individual patients within tribal clinic and Indian Health Service settings. Few, if any, of these programs have intervened broadly at environmental or policy levels [10]. Reasons for the lack of environmental and policy interventions vary but include limited AI health outcomes data [11, 12], which hinders tribal leaders in developing effective health policies. Additionally, public databases that catalog and monitor federal and state policies do not include the policies of sovereign tribal nations within these databases [13, 14]; thus, health planners and researchers remain unfamiliar with tribal policies as well as tribal policymaking processes. In terms of environmental interventions, most evidence-based environmental interventions, such as the implementation of sidewalks and streetlights to promote physical activity or healthy retail corner store strategies to promote healthy eating, have been designed primary for urban settings, with few or none having been tailored to and evaluated in sovereign tribal lands.

The Tribal Health and Resilience in Vulnerable Environments or “THRIVE” study is a community-based participatory research (CBPR) study aimed at improving the food environments within the Chickasaw Nation and the Choctaw Nation of Oklahoma. To inform the development of appropriate interventions in each of these nations, our tribal and academic partners collaboratively developed and conducted several studies: (1) a cross-sectional survey of food and activity environments, health behaviors, and health outcomes, (2) a health impact assessment, and (3) qualitative research with tribal convenience store employees and patrons. Here, we present data from the first of these studies to analyze the prevalence and associations between selected CVD risk factors (i.e., tobacco use, physical activity, and vegetable and fruit intake) and health outcomes (type 2 diabetes, obesity, and hypertension) for the two nations. Study findings were used to identify and prioritize evidence-based community-driven, multilevel intervention strategies to improve health and well-being in both tribal nations.

## Methods

### Study Orientation and Partnership

A CBPR orientation guided all aspects of this study. Our tribal-university partnership was comprised of university researchers with prior experience conducting CBPR and key stakeholders from both nations who represented a range of tribal service sectors, including health promotion, nutritional and clinical services, and tribal Institutional Review Boards (IRB) and research divisions. The development of our partnership processes and study methods was informed by partners’ previous CBPR with tribes in Oklahoma and elsewhere [15]. A steering committee, comprised of 15 partners, met in person monthly or more often throughout the study at tribal facilities. To inform the development of this baseline survey, the study PI, a tribal citizen, and the lead author of this manuscript presented key questions from national health surveys, such as BRFSS as well as surveys from studies conducted with other AI communities. Survey questions were discussed and selected based on existing standardized questionnaires, modified slightly in language, to be implemented within these tribal communities. This study was reviewed and approved by the Institutional Review Boards of the University of Oklahoma as well as the Chickasaw Nation and Choctaw Nation of Oklahoma. Per the preferences of the tribal partners tribal identity are not included when presenting specific survey results.

### Setting

The Chickasaw Nation and the Choctaw Nation of Oklahoma, both located in the southeastern portion of Oklahoma, are similar in size, population, and rurality. The Chickasaw Nation occupies 7270 mile^2^ (~10% of the area of Oklahoma), with a total population of 277,416, of whom 32,312 (12%) are AI [16]. The Choctaw Nation of Oklahoma occupies 10,602 mile^2^ (~15% of the area of Oklahoma), with a total population of 224,472, of whom 39,984 (18%) are AI. These tribal nations, once reservations, are now classified as Tribal Jurisdictional Areas by the US government. Poverty rates for all residents of the Chickasaw Nation (15.3%) and the Choctaw Nation of Oklahoma (20.7%) exceed the national poverty rate (13.8%) [16].

### Study Recruitment and Data Collection

Potential study participants were recruited and screened for eligibility by trained tribal staff in community locations selected by tribal research partners. These locations included tribal community centers, health clinics, tribal convenience stores, health fairs, and at community events including tribal cultural fairs and festivals. To be eligible, participants had to be at least 18 years old, live within the jurisdictional areas of the two tribal nations, and self-identify as American Indian or Alaskan Native. Participants elected whether they wanted to complete a paper survey or an electronic survey via an iPad. All participants completed informed consent prior to beginning the survey and were mailed a $30 gift card afterward. A total of 513 AI adults completed the survey between April and May of 2015 with a survey response of 91.4%.

### Measures

#### Health Outcomes

We assessed diabetes and hypertension by asking “Have you ever been told by a doctor, nurse, or other health professional that you have diabetes/high blood pressure?” Response options included Yes, Yes but I am female and was told only during pregnancy, No, and told that I was borderline diabetic/pre-hypertensive. Only “Yes” responses were considered endorsement of the health condition. Obesity was determined from body mass index calculated from self-reported height and weight. Body mass index ≥30 kg/m^2^ was considered obese.

#### Risk Factors

We assessed tobacco use with the question, “How often do you currently use tobacco products (i.e. smoking, dipping, etc.)?” Participants were considered to use tobacco if they responded “Daily”, “Several times a week”, “Only occasionally”, or “Monthly”; those who responded “I don’t use tobacco products” were coded as nonusers. We determined physical activity level by asking “How many days a week do you usually do at least 30 minutes of physical activity or walking that increases your heart rate or makes you breath harder than normal? (e.g. mowing the lawn, carrying light loads, bicycling at a regular pace, jogging, etc.)”. Low physical activity was defined as <5 days a week, since 5 or more days a week would be at least 150 min per week (30 min × 5 days) of physical activity, which is the national recommendation [17]. Fruit and vegetable intake was measured separately and was determined by asking two separate questions: “About how many servings of [fruit/vegetable] (include 100% pure [fruit/vegetable] juice) do you eat or drink each day?” Several examples for serving size were provided, and participants who responded <2 servings or <3 servings were defined as low fruit or low vegetable intake, respectively [18].

#### Demographic Characteristics

We determined age by asking “How old are you?” and categorized responses as 18–24, 25–44, 45–64, and 65+ years. Education was assessed with the question “What is the highest level of education you have completed?” For employment, we asked “Which of the following best describes your current, main daily activities and/or responsibilities?” Household income was assessed by asking “What was the total combined income of your household in the past year, including income from all sources, such as wages, salaries, Social Security or retirement benefits, help from relatives and so forth? Please choose total income before taxes.”

#### Statistical Analysis

We used logistic regression and marginal standardization to calculate adjusted prevalence estimates for health outcomes and risk factors [19]. Models were adjusted for age, sex, marital status, education, employment, and study site (i.e., Nation A and Nation B). We did not include income as an adjustment variable due to missing data. The same method was used to calculate prevalence ratios and 95% confidence intervals to examine the association between CVD-related risk factors and health outcomes. Analyses were performed from July through August 2015 using Stata 12 (StataCorp LP, College Station TX, 2014).

## Results

Nearly half of participants were aged 25–44 (46%), while another third were aged 45–64 years (35%) (Table 1). The majority of participants were female (75%), married or living with a partner (60%), and had completed at least some post-secondary education (64%). Most participants were employed (77%), and 57% had an average annual household income below $40,000. Demographic characteristics were similar in both sites.

**Table 1:** Demographic characteristics among American Indians from two tribal communities in Oklahoma

Characteristic	All participants (*n* = 513)	Nation A (*n* = 259)	Nation B (*n* = 254)
	%	%	%
Age, years			
18–24	9	9	10
25–44	46	48	44
45–64	35	33	37
65+	10	11	9
Female	75	76	74
Married or living with partner	60	63	57
Completed education			
<High school graduate	6	5	7
High school graduate/GED	30	34	26
Some college or some technical school	30	27	33
Associate’s degree or technical college degree	21	24	17
4-year college degree or higher	14	10	17
Employed full- or part-time	77	74	80
Household income in the past year			
<$20,000	26	27	26
$20,000–39,999	31	32	30
$40,000–79,999	27	24	30
$80,000+	16	17	14

Number of missing values: age *n* = 25, marital status *n* = 4, education *n* = 2, employment *n* = 2, income *n* = 4

Adjusted prevalences for CVD-related health outcomes and risk factors are presented in Table 2. The overall prevalence of diabetes (25%), obesity (56%), and hypertension (48%) was high. All three health outcomes were more prevalent among participants who were older, not currently married or living with a partner, had less education, and not employed. The prevalence of diabetes and obesity was similar in males and females, while the prevalence of hypertension was higher in males than females (58 vs. 45%). The prevalence of obesity was higher in Nation A compared to Nation B (58 vs. 53%), but the prevalence of diabetes and hypertension was similar in both study sites.

**Table 2:** Adjusted prevalence of health outcomes and cardiovascular disease risk factors among American Indians from two tribal communities in Oklahoma (*n* = 513)

	Health outcomes								Risk factors					
	Diabetes		Obesity		Hypertension		Tobacco use		Low physical activity^a^		Low fruit intake^b^		Low vegetable intake^c^	
	%	95% CI	%	95% CI	%	95% CI	%	95% CI	%	95% CI	%	95% CI	%	95% CI
Overall	25	21–28	56	51–60	48	44–52	26	22–29	71	67–75	56	51–60	75	71–79
Age, years														
18–24	2	0–6	21	9–33	8	1–16	24	12–36	68	55–82	62	48–77	79	67–90
25–44	14	9–19	61	54–67	37	30–43	31	25–38	74	68–80	55	48–62	79	73–84
45–64	39	32–47	60	52–67	66	59–74	25	19–32	66	59–73	58	51–66	73	66–79
65+	38	23–52	50	34–65	79	66–93	8	7–15	81	70–91	42	27–57	60	45–76
Sex														
Female	25	21–29	56	51–61	45	40–50	23	19–27	77	73–82	56	51–61	74	69–78
Male	24	17–32	53	44–62	58	50–66	34	25–42	53	45–62	55	46–64	79	71–86
Marital status														
Never married, div/sep/wid	29	23–35	62	55–68	52	45–58	25	19–31	68	62–74	52	45–59	70	64–77
Married or living with partner	21	17–26	52	46–57	46	41–51	26	21–31	74	69–79	58	52–64	78	73–83
Completed education														
<High school graduate	28	13–43	57	38–76	55	37–74	39	20–58	62	44–80	55	36–74	77	61–94
High school graduate/GED	28	21–35	64	56–72	56	48–63	34	26–42	69	61–76	55	47–63	78	71–85
Some college or some technical school	22	16–29	54	46–62	48	41–55	30	23–37	76	69–82	60	52–68	71	64–79
Associate’s or tech college degree	21	13–29	54	44–64	43	34–52	11	5–18	76	67–84	50	40–60	74	65–83
4-year college degree or higher	24	14–34	43	31–55	38	28–49	12	4–20	66	54–77	55	43–68	76	66–87
Employment status														
Not employed^d^	31	23–39	64	55–74	54	44–63	31	22–40	67	58–76	55	46–65	82	74–89
Employed full- or part-time	22	18–26	53	48–58	47	42–51	24	20–28	73	68–77	56	50–61	72	68–77
Site														
Nation A	25	20–30	58	52–64	48	42–53	25	20–31	78	73–93	64	58–70	80	75–85
Nation B	24	19–29	53	47–60	49	43–55	26	20–31	64	58–71	46	40–53	70	64–76

Adjusted for age, sex, marital status, education, employment, and site

^a^<5 days/week of at least 30 min of physical activity per day

^b^<2 servings/day

^c^<3 servings/day

^d^Unemployed, student, homemaker, disabled, and retired

The prevalence of tobacco use was 26% (Table 2). Tobacco use was the highest among participants aged 25–44 years (31%), males (34%), and those who had a high school education or less (34–39%). The majority of participants reported low physical activity (71%). Low physical activity was the most prevalent among participants aged 65 years and greater (81%), females (77%), and Nation A participants (78%). The overall prevalence of low fruit intake was 56%. Low fruit intake was more likely among the youngest participants aged 18–24 years (62%) and Nation A participants (64%). Three quarters (75%) of participants reported low vegetable intake. Low vegetable intake was the highest among those who were not employed (82%) and Nation A participants (80%).

The prevalence of diabetes, obesity, and hypertension tended to be higher among participants who reported any tobacco use compared to those who did not, but these findings were not statistically significant (Table 3). The prevalences of these health outcomes were higher among participants with low physical activity levels compared to recommended physical activity levels. This was particularly evident for diabetes (PR 2.1, 95% CI 1.4–3.2, *p* = 0.01) and obesity (PR 1.5, 95% CI 1.2–1.8, *p* < 0.01). We found little association between low fruit and low vegetable intake and the health outcomes.

**Table 3:** Adjusted association between cardiovascular disease risk factors and health outcomes among American Indians from two tribal communities in Oklahoma

	Diabetes		Obesity		Hypertension	
	PR	95% CI	PR	95% CI	PR	95% CI
Use of any tobacco products	1.2	0.8–1.7	1.0	0.9–1.2	1.1	0.9–1.4
Low physical activity^a^	2.1	1.4–3.2	1.5	1.2–1.8	1.1	0.9–1.3
Low fruit intake^b^	1.2	0.9–1.6	1.1	0.9–1.3	1.0	0.8–1.1
Low vegetable intake^c^	1.0	0.7–1.4	1.1	0.9–1.3	0.9	0.7–1.0

Adjusted for age, sex, marital status, education, employment, and site

*PR* prevalence ratio

^a^<5 days/week of at least 30 min of physical activity per day

^b^<2 servings/day

^c^<3 servings/day

## Discussion

We found high rates of diabetes, obesity, and hypertension within both tribal nations as well CVD-related risk factors (i.e., tobacco use, low physical activity, and low vegetable and/or fruit intake). Our findings are consistent with the previous studies that showed that the prevalence of diabetes, obesity, and hypertension is higher among AIs than other racial/ethnic groups [1, 20–22]. For instance, a recent analysis of National Health Interview Survey data found that, between 2004 and 2008, 39.4% of AIs were obese compared with 24.3% of non-Hispanic whites, and 17.5% of AIs were diabetic compared to 6.6% of non-Hispanic whites [20]. Similarly, studies examining national BRFSS data, including our own analysis, have identified higher rates of obesity and diabetes for AIs (31 and 12.4%) compared to respondents from all other racial/ethnic groups (20.9 and 6%), and that these rates have steadily increased over the last two decades even as diabetes rates among non-Hispanic whites have remained relatively stable [1, 21, 22]. Data from the Oklahoma BRFSS reported that the prevalence of obesity and diabetes among Oklahoma AIs was even higher (42 and 15%), while our current findings suggest that the prevalence of obesity (56%) and diabetes (25%) may be higher still in the most rural of tribal communities in Oklahoma.

The prevalence of hypertension in our study was 48%. The Centers for Disease Control and Prevention (CDC) and Racial and Ethnic Approaches to Community Health (REACH 2010) initiative, which included a random cross-sectional survey of communities participating in REACH, showed that AIs had a higher prevalence of obesity (37.7%), diabetes (19.7%), and hypertension (36.8%) compared to any other racial/ethnic group [23]. Further, AI adults were found to have the highest prevalence of these combined risk factors for CVD, with >80% having one or more (88.2% men, 82.8% women) and over a third with three or more (35.7% men, 33.3% women) [23].

In our current study, AIs reported very low fruit and vegetable intake. These findings are consistent with the Strong Heart Study, and others reported that the diet in many AI communities is low in fruits and vegetables, high in fat, and below the Recommended Dietary Allowances for several vitamins and minerals, including calcium and iron [24, 25]. Surprisingly, we found no association between fruit and vegetable consumption and the selected health outcomes. One possible reason for this could be limitations of the two-question short assessment we administered. While we sought to administer a measure that limited respondent burden, a more comprehensive dietary measure may have yielded different results. Additionally, evidence suggests that incorporating elements of cultural and racial food preferences as well as geographic region may further refine standard dietary measurements to be more accurate in diverse populations [26].

Additionally, 71% of participants in our study reported low physical activity, consistent with other studies that have reported that most AI adults, and more than three quarters of AI youth, do not meet minimum public health recommendations for physical activity [27, 28], even though some evidence suggests that AIs engage in physical activity at higher rates than other racial/ethnic groups [29]. Finally, 25% of individuals we surveyed reported using tobacco. Data from the 2014 National Health Interview Survey reported that 29.2% of AIs smoked, nearly double the rate found in the US general population (16.8%), and the highest of any racial/ethnic group [30].

Findings from our study support existing literature that CVD-related health disparities persist among AIs despite the implementation of universal policy approaches, such as smoking bans or comprehensive health care reform via the Affordable Care Act [10]. For example, between 2005 and 2014, smoking rates declined within the US general population by nearly 20%, while the reduction among AIs was less than 8% [30]. The National Healthcare Disparity Annual Report, issued by the Agency for Healthcare Research and Quality, found that in 2015, AIs had worse access to care than whites for 30% of access measures and received worse care than whites for about 40% of quality measures [31]. The report also found that disparities are not narrowing with improvements in health care [31].

Indeed, efforts to make meaningful progress in eliminating AI health disparities must address several key issues. First, AIs must be regularly included in national health surveys. The exclusion of AIs due to small sample sizes hinders efforts to monitor AI health status and identify important geographic and sociodemographic differences in health outcomes that exist within and across this diverse population [1, 11, 12]. Regular tracking of AI health behaviors and risk factors will support tribal leaders to make informed policy decisions to address and eliminate disparities.

Additionally, despite the widespread promotion of health enhancing environmental and policy interventions (e.g., menu labels, bike lanes, food pricing), their generalizability to tribal communities and settings remains unclear [32–35]. Tribal policymaking processes often remain poorly understood by researchers and health planners alike, hindering the evaluation and scale-up of effective health promotion interventions and widening disparities. Research efforts on state and national health promotion policies and determinants of policy adoption must include tribal policies within their analyses. Finally, efforts to intervene upon the “root causes” of AI health disparities—poor housing, unemployment, and limited or no access to healthy foods—via multilevel interventions that address the social and political contexts creating and maintaining AI health inequities must be prioritized.

To that end, findings from this survey, as well as other data collected as part of this study, were presented back to community members and tribal leaders to develop environmental interventions to address CVD and diabetes. Interventions to improve both the food and physical activity environments were identified. Ultimately, the tribal food environments, specifically tribally owned and operated convenience stores, were prioritized. We selected these environments within which to intervene because the tribes had full authority in these settings to make environmental and policy changes. We also sought to capitalize on the growing momentum within both nations, identified through 12 focus groups we conducted, for greater community access to healthy and affordable foods. Therefore, we randomized eight tribally owned convenience stores (four controls and four interventions) and implemented “healthy makeovers” within the stores consisting of: (1) increased availability and convenience of healthy foods, (2) reduced pricing for healthy foods, and (3) the promotion and marketing of these foods within the tribal stores. The intervention and its trial, now underway, are part of a new “Health in All Policymaking” approach initiated by both nations. With the ultimate aim of disseminating findings and lessons learned to tribal policymakers within and beyond Oklahoma, our tribal-academic partnership is using best practice recommendations for the synchronous implementation and research, “learning and doing,” and assessing the health impact and cost-effectiveness of our intervention strategies. In doing so, we hope to facilitate the future scale-up of promising practices that will lead to sustained improvements in the risk factors and health outcomes examined in the current study as well as others critical to the health and well-being of American Indian communities.

### Limitations

This study has several limitations. Although efforts were made to select a diverse sample of AIs across the two tribal nations, this was not a random sample. Thus, our findings are limited in their generalizability even within the tribal nations in which they were collected. Similarly, our sample was overwhelmingly female. Due to missing data, we were not able to control for income. We instead included employment and education in our adjusted models. Additionally, while we examined a comprehensive set of risk factors and health outcomes, all data were self-reported and may not be accurate assessments of health behavior or health status. We do, however, present prevalences and adjusted ORs from multiple logistic regression models that controlled for confounding factors, and the findings of our study are consistent with those of other studies examining CVD-related health outcomes among AIs. Further, our study contributes to the gap in existing literature regarding CVD-related health outcomes.

## Conclusion

The AI population is regularly excluded from national health surveys due to small sample sizes.

Research to address CVD-related disparities among AIs is seriously hampered by researchers’ and health planners’ unfamiliarity with the tribal policymaking process and the absence of tested strategies to engage tribal leaders the development of policy and environmental interventions. As a result, the feasibility of implementing evidence-based policy and environmental interventions to address such disparities has not been adequately examined within the diverse sociocultural and political contexts of sovereign tribal nations. Multilevel interventions that address the social determinants of these and other disparities as well as the development of strategies for the scale-up and implementation of interventions shown to be effective within AI communities are critical to the elimination of persistent AI health disparities.
